# Recent structural insights into the mechanism of lysozyme hydrolysis

**DOI:** 10.1107/S2059798321000346

**Published:** 2021-02-19

**Authors:** Ichiro Tanaka, Ryota Nishinomiya, Ryosuke Goto, Shun Shimazaki, Toshiyuki Chatake

**Affiliations:** a Graduate School of Science and Engineering, Ibaraki University, Hitachi, Ibaraki 316-8511, Japan; b Frontier Research Center for Applied Atomic Sciences, Ibaraki University, Tokai, Ibaraki 319-1106, Japan; c College of Engineering, Ibaraki University, Hitachi, Ibaraki 316-8511, Japan; d Institute for Integrated Radiation and Nuclear Science, Kyoto University, Kumatori, Osaka 590-0494, Japan

**Keywords:** lysozyme hydrolysis, hydrogen-bond network, high-resolution X-ray diffraction, neutron diffraction

## Abstract

Lysozyme hydrolyzes the glycosidic bonds between *N*-acetylmuramic acid and *N*-acetylglucosamine in peptidoglycans located in the bacterial cell wall. The mechanism of the hydrolysis reaction of lysozyme was first studied more than 50 years ago; however, it has not yet been fully elucidated and various mechanisms are still being investigated. One reaction system that has commonly been proposed is that the lysozyme intermediate undergoes covalent ligand binding during hydrolysis. However, these findings resulted from experiments performed under laboratory conditions using fluorine-based ligands, which facilitate the formation of covalent bonds between the ligands and the catalytic side chain of lysozyme. More recently, high-resolution X-ray structural analysis was used to study the complex of lysozyme with an *N*-acetylglucosamine tetramer. As a result, the carboxyl group of Asp52 was found to form a relatively strong hydrogen-bond network and had difficulty binding covalently to C1 of the carbohydrate ring. To confirm this hydrogen-bond network, neutron test measurements were successfully performed to a resolution of better than 1.9 Å.

## Abstract 2

The complex of lysozyme with an *N*-acetylglucosamine tetramer shows a relatively strong hydrogen-bond network around a catalytic residue via high-resolution X-ray structural analysis. This indicates a potentially different hydrolysis mechanism to that through a glycosyl intermediate, and this is expected to be proved using neutron experiments.

## 1. Introduction   

Lysozyme is a well known enzyme that hydrolyzes the glycosidic bonds between *N*-acetylmuramic acid and *N*-acetyl­glucosamine in peptidoglycans located in the bacterial cell wall. After the binding of the carbohydrate ring hexamer to the catalytic site of lysozyme, hydrolysis occurs between the fourth and fifth carbohydrate rings via two catalytic amino acids, such as Glu35 and Asp52 in the case of hen egg-white (HEW) lysozyme. Although the mechanism of the hydrolysis reaction of lysozyme was first investigated over 50 years ago (Phillips, 1967 ▸; Koshland, 1953 ▸; Imoto *et al.*, 1972 ▸), a number of potential mechanisms are actively being studied. The reaction system that has most commonly been proposed is that the intermediate of lysozyme forms a covalent bond between the fourth carbohydrate ring and the side chain of Asp52 during hydrolysis (Vocadlo *et al.*, 2001 ▸). However, these findings were obtained under laboratory conditions using fluorine-based ligands, which facilitate the formation of covalent bonds to the catalytic side chain of lysozyme. Furthermore, a shift of the carbohydrate chain to Asp52 was expected owing to distortion of the carbohydrate ring, forming a covalent bond through rotation of the side chain of Asp52. Many other structural analyses of lysozyme–carbohydrate complexes have been performed, such as complexes with tetra-*N*-acetylchito­tetraose or a tetramer of *N*-acetyl-d-glucosamine [(GlcNAc)_4_] (Maenaka *et al.*, 1995 ▸; Yamada *et al.*, 2015 ▸), as well as a complex with tri-*N*-acetylchitotetraose (Cheetham *et al.*, 1992 ▸) and with a trimer of *N*-acetyl-d-glucosamine (GlcNAc)_3_ with moranoline at the end (Ogata *et al.*, 2013 ▸). However, there is currently no clear evidence of covalent bonding between the fourth carbohydrate ring of (GlcNAc)_4_ (NAG4) and the side chain of Asp52 during hydrolysis under natural conditions, whereas particular substrate analogs and Asp52 may easily form covalent bonds in an artificial manner. In addition, research has yet to consider the formation of a hydrogen-bond network around the catalytic amino acids using high-resolution data. Furthermore, turnover-rate experiments on mutated lysozyme indicated that most of the hydrolysis reaction did not take place via glycosyl formation (Abe *et al.*, 2016 ▸).

The hydrogen-bond network needs to be characterized to determine whether or not rotation occurs. To this end, high-resolution X-ray and neutron diffraction are indispensable tools, the latter of which is one of the best and most powerful methods for determining protein structures, including H atoms, unambiguously (Niimura & Podjarny, 2011 ▸). Two neutron studies have investigated HEW lysozyme (Niimura *et al.*, 1997 ▸; Bon *et al.*, 1999 ▸); however, these did not contain ligands. Diffraction experiments using new-generation neutron sources can potentially characterize these structures (Tanaka *et al.*, 2020 ▸).

In this study, the hydrogen-bond networks of a lysozyme–carbohydrate complex were analyzed using high-resolution X-rays to investigate and confirm the hydrolysis mechanism of this complex. Neutron test experiments were also performed to confirm the resolution of data from large crystals grown in D_2_O buffer. Experiments were conducted at the optimal pH (∼5) of the enzyme using natural enzymatic products [(GlcNAc)_4_ as a product from (GlcNAc)_6_] to provide a snapshot of the catalytic reaction intermediates.

## 2. Materials and methods   

### 2.1. Sample crystallization for X-ray and neutron diffraction   

HEW lysozyme was purchased from Sigma–Aldrich, St Louis, Missouri, USA (catalog No. L6876). (GlcNAc)_3_ and (GlcNAc)_4_ were purchased from Dextra Laboratories, Reading, England (catalog Nos. C8003 and C8004, respectively). (GlcNAc)_4_ was also purchased from Megazyme, Bray, Ireland (catalog No. O-CHI4). The proteins and carbo­hydrates were used without further purification for X-ray structural analysis. Protein solutions were filtered using a 0.22 µm filter to remove any impurities.

For X-ray diffraction, three types of crystals were used. A lysozyme–carbohydrate complex crystal was obtained in H_2_O from 6 µl of a solution consisting of 40 mg ml^−1^ lysozyme, 2.1 mg ml^−1^ (GlcNAc)_4_ and 0.7 *M* NaCl in 50 m*M* sodium acetate pH 4.5 buffer using the micro-batch method at 293 K. A lysozyme–carbohydrate complex crystal was obtained in D_2_O from a mixture of 5 µl 40 mg ml^−1^ lysozyme containing 2.1 mg ml^−1^ (GlcNAc)_4_ and 5 µl 0.7 *M* NaCl in a D_2_O buffer of 50 m*M* sodium acetate pD 4.5 by the hanging-drop method at 293 K. The reservoir used was 0.7 *M* NaCl in a D_2_O buffer of 50 m*M* sodium acetate pD 4.5. A crystal of lysozyme without carbohydrate was obtained in H_2_O from 6 µl of a solution consisting of 15 mg ml^−1^ lysozyme and 0.7 *M* NaCl in 50 m*M* sodium acetate pH 4.5 using the micro-batch method at 293 K.

For neutron diffraction, two complexes were prepared. A lysozyme–(GlcNAc)_4_ complex crystal was obtained from 20 µl of a solution consisting of 15 mg ml^−1^ lysozyme, 1.0 mg ml^−1^ (GlcNAc)_4_, 0.25 *M* NaCl in a D_2_O buffer of 50 m*M* sodium acetate pD 4.5 by the sitting-drop method using 1 ml reservoir solution consisting of 0.5 *M* NaCl in the same buffer at 293 K. A lysozyme–(GlcNAc)_3_ complex crystal was obtained from 10 µl of a solution consisting of 17.5 mg ml^−1^ lysozyme, 0.77 mg ml^−1^ (GlcNAc)_3_, 0.6 *M* NaCl in a D_2_O buffer of 50 m*M* sodium acetate pD 4.5 by the sitting-drop method with 0.5 ml reservoir solution consisting of 1.2 *M* NaCl in the same buffer at 293 K.

### 2.2. X-ray diffraction experiments and analysis   

Prior to conducting the X-ray diffraction experiments, the crystals were flash-cooled in liquid nitrogen after soaking in 30%(*v*/*v*) glycerol buffer. The crystals were irradiated with X-rays at a synchrotron facility (BL-5A at Photon Factory, KEK, Japan) under nitrogen gas at 100 K and a full data set was collected. *HKL*-2000 (Otwinowski & Minor, 1997 ▸) or *XDS* (Kabsch, 2010 ▸) were used to reduce the raw data, and *Phenix* (Liebschner *et al.*, 2019 ▸) and *Coot* (Emsley *et al.*, 2010 ▸) were used to analyze the structures. PDB entry 4wld (Yamada *et al.*, 2015 ▸) was used as the initial model. The atoms were refined using the weight-optimization function included in *phenix.refine*, while anisotropic temperature factors were calculated. Figures were created using *PyMOL* (*PyMOL Molecular Graphics System* version 2.4; Schrödinger). The experimental conditions, data-reduction statistics and analyses are given in Table 1 ▸. The atomic coordinates and structural factors of the lysozyme complex in H_2_O, the lysozyme complex in D_2_O and the lysozyme-only sample have been deposited in the Protein Data Bank as entries 7br5, 7deq and 7der, respectively.

With regard to the top difference-map peaks in the three models, we note the following: for the complex in H_2_O there is evidence of X-ray radiation damage to the Cys6–Cys127 disulfide, *i.e.* some movement of the S atoms in the disulfide, for the complex in D_2_O there is disorder at Arg128 that is not easy to model and is not pertinent to the subject of the study, and for lysozyme alone there is disorder in the sodium site environment.

### 2.3. Neutron diffraction experiments   

**Figure 1:**
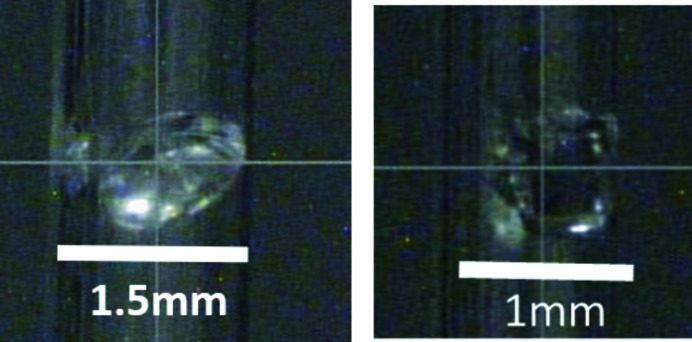
Single crystals of the lysozyme–(GlcNAc)_3_ complex (left; 1.1 × 0.65 × 0.4 mm, 0.29 mm^3^ volume) and the lysozyme–(GlcNAc)_4_ complex (right; 0.7 × 0.7 × 0.25 mm, 0.12 mm^3^ volume) used in neutron diffraction experiments.

Prior to conducting the neutron diffraction experiments, each crystal was placed in a quartz capillary after removing the buffer liquid from the crystal surface. The capillaries were sealed with a small amount of mother liquor to prevent the crystal from drying. The single crystals of the lysozyme–(GlcNAc)_3_ complex and the lysozyme–(GlcNAc)_4_ complex are shown in Fig. 1 ▸. The neutron experiments were conducted on BL03 (iBIX; Tanaka *et al.*, 2010 ▸) of the Material and Life Science Facility (MLF) of J-PARC (Japan Proton Accelerator Research Complex) at 298 K with 400 kW proton power for 14 h in the case of the (GlcNAc)_3_ complex and 500 kW proton power for 8 h in the case of the (GlcNAc)_4_ complex. The beam cross section was 3 mm in diameter with a wavelength range of 2.18–6.18 Å.

## 3. Results and discussion   

### 3.1. X-ray structural analysis   

**Figure 2:**
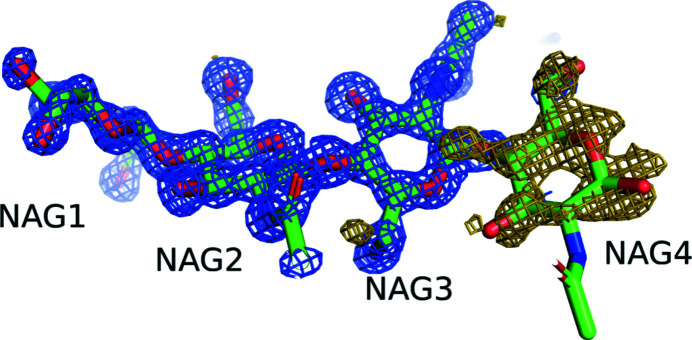
Stick structure of (GlcNAc)_4_ bound to lysozyme in D_2_O with electron-density maps (green, carbon; red, oxygen; blue, nitrogen). NAG1, NAG2, NAG3 and NAG4 correspond to the first, second, third and fourth residue of (GlcNAc)_4_, respectively. The blue and olive electron-density maps are 2*F*_o_ − *F*_c_ at 1.5σ and *F*_o_ − *F*_c_ at 3.0σ, respectively, which were drawn by omitting NAG4.

**Figure 3:**
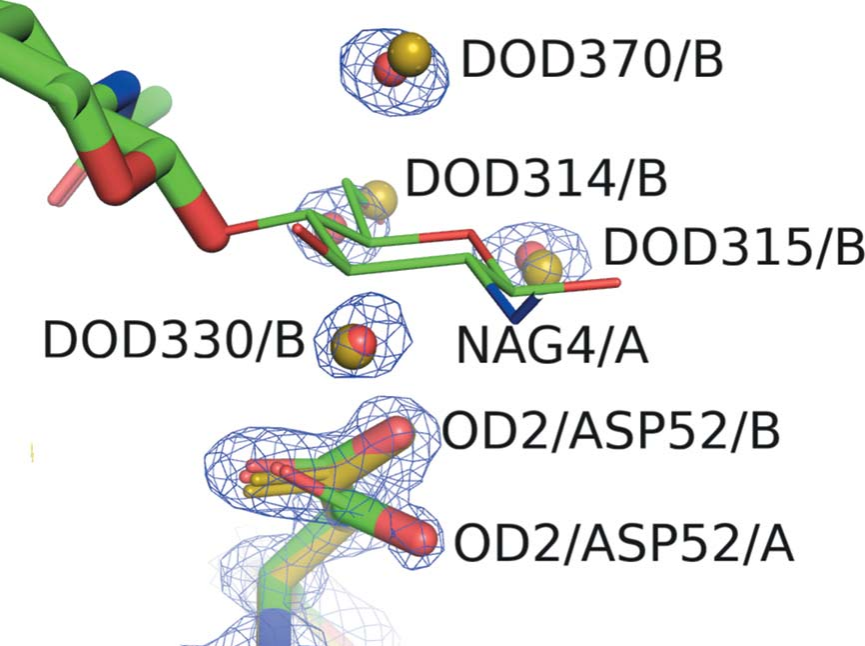
Superimposed stick structures of the lysozyme–(GlcNAc)_4_ complex (CPK colors with green for carbon; complex in D_2_O) and of lysozyme without ligands (olive; lysozyme alone) where only NAG4 is drawn as a wire model for clarity. O atoms of water molecules both in lysozyme alone and in the complex in D_2_O are illustrated as olive and red balls, respectively. NAG4/A and OD2/ASP52/A are in the A conformation. In contrast, four water molecules and OD2/ASP52/B are located in the B conformation. OD2 of Asp52 in lysozyme alone overlaps with OD2/ASP52/B. The blue contour lines correspond to 2*F*_o_ − *F*_c_ maps at 1.5σ for the complex in D_2_O, except for (GlcNAc)_4_.

The crystal structures of two complexes in D_2_O and H_2_O and of lysozyme alone in H_2_O were determined using high-resolution data at 1.0 to 1.03 Å. Since there were no significant differences between the (GlcNAc)_4_ complex in D_2_O and in H_2_O, the D_2_O complex structure will be referred to as the complex structure from this point in the discussion. At the ligand-binding site, (GlcNAc)_4_ was confirmed as a tetramer of GlcNAc (Fig. 2 ▸). Although the occupancy of the fourth residue of (GlcNAc)_4_ (NAG4) was found to be relatively low (0.36 and 0.21 for the complexes in D_2_O and H_2_O, respectively) compared with the other three NAGs, NAG4 can be readily observed in the 3σ difference Fourier map in Fig. 2 ▸. In addition, NAG4 and four water molecules share the same site, with double conformations A and B with occupancies of 0.36 and 0.64, respectively (Fig. 3 ▸). The four water molecules are well conserved among the structures of the complexes and of lysozyme alone.

When comparing the structure around the D-site carbo­hydrate (NAG4), four water molecules in the complex overlap with those in the structure without ligand, and these water molecules and NAG4 share a similar site with double conformations as described previously. The catalytic residue Asp52 in the complex structure has also double conformations A and B, with occupancies of 0.44 and 0.56, respectively. The corresponding residue of lysozyme alone overlaps with the B conformation of the complex, which is closer to the NAG4 site (Fig. 3 ▸). Based on these findings, approximately 40% of the crystal molecules have NAG4 and Asp52 orientations that are distant from the NAG4 site (A conformation) due to ligand binding. NAG4 may be partly hydrolyzed during the crystallization process.

**Figure 4:**
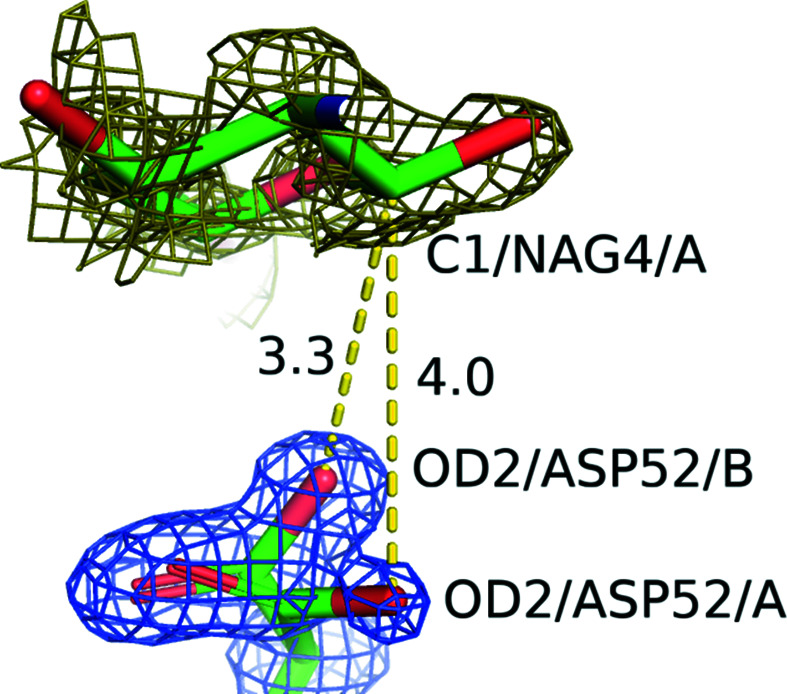
The distances (labeled in Å) between C1/NAG4/A and OD2/ASP52 in the A and B conformations are shown as yellow dashed lines. The blue and olive contour lines represent 2*F*_o_ − *F*_c_ maps at 1.5σ and 0.6σ for the complex in D_2_O, respectively. Atoms are shown as sticks (green, carbon; red, oxygen; blue, nitrogen).

The distance between C1 of NAG4 and OD2 of Asp52 was examined. The distances are 4.0 and 3.3 Å for the A conformation, where the carbohydrate was bound to the D site, and for the B conformation, where no ligand was bound, respectively (Fig. 4 ▸). The distance appears to be too great for Asp52 to bind to C1 of NAG4; it can be thought that the A conformation is close to the hydrolysis intermediate.

**Figure 5:**
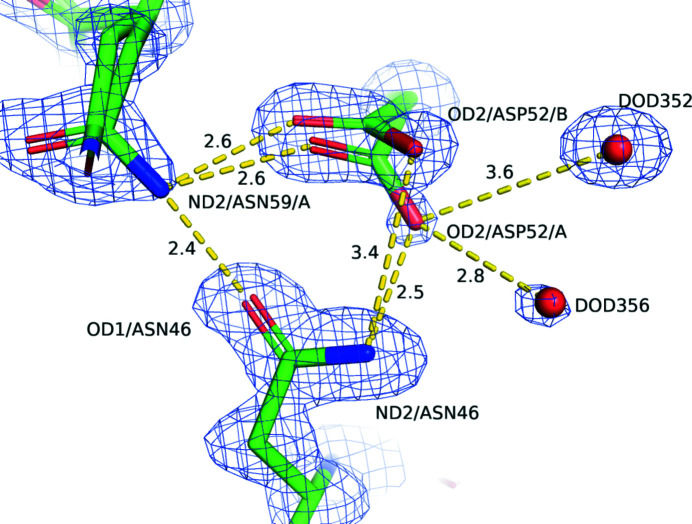
Hydrogen-bond network around Asp52. Yellow dashed lines represent hydrogen bonds or other interactions. The numbers denote the distances in Å. Atoms are shown as sticks (green, carbon; red, oxygen; blue: nitrogen). The O atom of the water molecule is shown as a red sphere. The electron-density maps are shown as blue contour lines for the protein (2*F*_o_ − *F*_c_ map at 1.5σ).

The hydrogen-bond network around Asp52 was also examined (Fig. 5 ▸). A relatively solid hydrogen-bonding polygon is formed by Asp52 and Asn59 in the A conformation and Asn46. The hydrogen bonds, with distances of 2.2–2.6 Å, can be categorized as moderately strong (Jeffery, 1997 ▸). Additionally, OD2 of Asp52 makes bifurcated hydrogen bonds to DOD356 and DOD352 with distances of 2.8 and 3.6 Å, respectively. Based on this, OD2 of Asp52 in the A conformation appears to have difficulty in forming a covalent bond to C1 of NAG4.

In the present complexes, two conformations are observed in the active site of the lysozyme molecule. The two conformations suggest that the present crystal structure may be a mixture of the lysozyme–first product (or new substrate) complex (A conformation) and the lysozyme–second product complex after the chemical reaction (B conformation). This is the first time that such a structure with co-existing conformations has been observed. This provides evidence that (GlcNAc)_4_ was hydrolyzed to (GlcNAc)_3_ at some stage.

In the present analysis, the native substrate is used. Interestingly, a covalent bond between lysozyme and (GlcNAc)_4_ is not observed, unlike in previous artificial complexes (Vocadlo *et al.*, 2001 ▸; Ogata *et al.*, 2013 ▸), suggesting the possibility of another hydrolysis mechanism differing from that via a glycosyl intermediate.

Instead of a covalent bond, the hydrogen-bond network differs between lysozyme complexes with the ligand and lysozyme without substrate. In order to obtain further detailed information, a neutron diffraction experiment is now needed to determine the hydrogen positions of the lysozyme, substrate and water molecules.

### 3.2. Neutron test experiment   

Prior to performing the neutron diffraction experiment, tests were conducted to determine whether the quality and volume of the crystals were sufficient for diffraction at a suitable resolution to observe the H atoms in the molecular structure. For both the lysozyme–(GlcNAc)_4_ and lysozyme–(GlcNAc)_3_ complexes, diffraction was successfully obtained to resolutions of 1.87 and 1.89 Å, respectively. At these resolutions, the orientations of the water molecules and the H atoms should be identifiable. For X-ray/neutron refinement, ambient temperature data need to be obtained using X-ray diffraction at a comparable resolution using the same crystals as those used for neutron full data measurements.

## 4. Conclusion   

A high-resolution structural analysis of lysozyme complexed with an *N*-acetylglucosamine tetramer [(GlcNAc)_4_] was successfully performed using X-ray diffraction. Compared with the structure of lysozyme alone, it was found that NAG4 shares its site with four water molecules that were well conserved in the structure of lysozyme alone. A relatively strong hydrogen-bond network was observed around the catalytic residues, specifically Asp52, and the distance between C1 of NAG4 and OD2 of Asp52 was observed to be 4.0 Å. These results indicate that it may be difficult for lysozyme to hydrolyze carbohydrate molecules via the glycosyl intermediate. To confirm the structure of this hydrogen-bond network, neutron diffraction test experiments were performed at resolutions that will permit us to observe the orientations of both water molecules and H atoms in a full neutron data collection.

## Supplementary Material

PDB reference: lysozyme, complex with (GlcNAc)_4_, obtained in H_2_O, 7br5

PDB reference: obtained in D_2_O, 7deq

PDB reference: without ligand, 7der

## Figures and Tables

**Table 1:** Experimental conditions, data-reduction statistics and analysis for X-ray diffraction experiments Values in parentheses are for the outermost shell.

PDB code	7br5	7deq	7der
Notes	Complex in H_2_O	Complex in D_2_O	No ligand in H_2_O
Diffraction source	BL-5A, Photon Factory	BL-5A, Photon Factory	BL-5A, Photon Factory
Wavelength (Å)	1.00	1.00	1.00
Temperature (K)	100	100	100
Space group	*P*4_3_2_1_2	*P*4_3_2_1_2	*P*4_3_2_1_2
*a*, *c* (Å)	77.309, 38.214	77.029, 38.285	78.777, 37.093
Resolution range (Å)	16.83–1.00 (1.036–1.000)	34.45–1.03 (1.067–1.030)	33.56–1.03 (1.067–1.030)
Total reflections	906663 (87281)	706539 (62633)	713001 (63054)
Unique reflections	61939 (6187)	57379 (5652)	58114 (5713)
Multiplicity	14.6 (14.1)	12.3 (11.1)	12.3 (11.0)
Completeness (%)	98.53 (99.95)	100.00 (100.00)	99.99 (100.00)
*R*_merge_	0.07595 (0.747)	0.06526 (1.274)	0.07402 (0.6635)
〈*I*/σ(*I*)〉	40.20 (4.38)	22.17 (2.21)	19.51 (2.25)
CC_1/2_	0.997 (0.893)	0.999 (0.837)	0.997 (0.929)
*R*_work_/*R*_free_	0.1392/0.1669	0.1325/0.1485	0.1487/0.1733
No. of non-H atoms			
Total	1343	1362	1285
Protein	1081	1078	1036
Ligand/ion	62	62	5
Water	193	210	233
*B* factor (Å^2^)			
Overall	18.00	16.90	17.70
Protein	15.10	13.80	14.70
Ligand/ion	20.70	18.00	17.10
Water	32.90	31.70	30.60
R.m.s.d. from ideality			
Bond lengths (Å)	0.006	0.006	0.006
Bond angles (°)	1.10	1.08	1.12
Ramachandran plot			
Most favored (%)	99	99	99
Allowed (%)	1	1	1
